# Dynamic Nature of Noncoding RNA Regulation of Adaptive Immune Response

**DOI:** 10.3390/ijms140917347

**Published:** 2013-08-22

**Authors:** Graziella Curtale, Franca Citarella

**Affiliations:** 1 Department of Medical Biotechnologies and Translational Medicine, University of Milan, Rozzano 20089, Italy; 2 Humanitas Clinical and Research Center, Rozzano 20089, Italy; 3 Department of Cellular Biotechnology and Hematology, Sapienza University of Rome, Rome 00185, Italy; E-Mail: citarella@bce.uniroma1.it

**Keywords:** noncoding RNAs, adaptive immunity, lymphocytes, epigenetic

## Abstract

Immune response plays a fundamental role in protecting the organism from infections; however, dysregulation often occurs and can be detrimental for the organism, leading to a variety of immune-mediated diseases. Recently our understanding of the molecular and cellular networks regulating the immune response, and, in particular, adaptive immunity, has improved dramatically. For many years, much of the focus has been on the study of protein regulators; nevertheless, recent evidence points to a fundamental role for specific classes of noncoding RNAs (ncRNAs) in regulating development, activation and homeostasis of the immune system. Although microRNAs (miRNAs) are the most comprehensive and well-studied, a number of reports suggest the exciting possibility that long ncRNAs (lncRNAs) could mediate host response and immune function. Finally, evidence is also accumulating that suggests a role for miRNAs and other small ncRNAs in autocrine, paracrine and exocrine signaling events, thus highlighting an elaborate network of regulatory interactions mediated by different classes of ncRNAs during immune response. This review will explore the multifaceted roles of ncRNAs in the adaptive immune response. In particular, we will focus on the well-established role of miRNAs and on the emerging role of lncRNAs and circulating ncRNAs, which all make indispensable contributions to the understanding of the multilayered modulation of the adaptive immune response.

## 1. Introduction

Adaptive immune response, generated by clonal selection of antigen-specific lymphocytes, provides an extremely versatile mechanism of host organism defense and increases its protection against subsequent exposure to the same antigen and/or reinfection with the same pathogen. Cells involved in the adaptive immune response are characterized by temporal development in primary lymphoid organs and functional differentiation in secondary lymphoid organs, phenomena whose successful regulation relies on a multilayered system of control [1,2]. In fact, it is now clear that the process of transcriptional regulation, carried out by general and specific transcription factors, must be framed in a picture in which the epigenetic control and the post-transcriptional control, which are also the result of non-protein coding RNA (ncRNA) activities, contribute in a fundamental manner. A growing number of observations indicate that different genome-encoded functional RNA species orchestrate the development and the differentiation state of complex tissues and specific cell types [3,4]. With regard to the adaptive immune response, while the regulative roles of several microRNAs (miRNAs) are well-established [5], recent, yet emerging, evidence demonstrates the relevance of a new heterogeneous repertoire of functional noncoding RNAs [6], which can be collectively referred to as long noncoding RNAs (lncRNAs).

The first and most generic association of miRNAs with the immune system was due to the detection of different miRNA expression profiles either in normal lymphocytes or lymphocytes derived from patients affected by immunological malignancies [7]. As a logical consequence of the identification of specific miRNA expression signatures, the next step has been the investigation of a possible causal relationship between miRNAs and the disease. Despite several *in vitro* studies identifying specific miRNA target genes involved in different aspects of cell biology, the first strong genetic evidence of the pivotal role of miRNAs in immune biology came from the analysis of mice knock-out for genes that are involved in the biogenesis of mature miRNAs, namely Dicer and Drosha. These studies established that miRNAs, as a whole, control the development and the homeostasis of the immune system [8–10]. Successively, it was established that the expression of several miRNAs is restricted to hematopoietic cells and is associated with specific developmental stages or functional differentiation of lymphocytes [11]. Moreover, it has been shown that miRNA concentration is dynamically modulated during the immune response, due to the existence of feed-back loops in which miRNAs control their own expression and, in so doing, contribute to the homeostasis of the immune system and the maintenance of cell identity [12,13]. In this context, uncovering the regulation of miRNA expression in the immune system became a major issue.

Genes coding for miRNAs are mostly transcribed by RNA polymerase II and, therefore, are subjected to the regulation of transcription factors, whose repertoire is different in different cells and whose activation can be regulated by extracellular stimuli. This, then, makes way for the transcriptional regulation of miRNAs by co-activators and co-repressors, including chromatin remodeling factors and other epigenetic factors. In addition, recent studies indicate that the expression of mature miRNAs in the cell can be controlled at a post-transcriptional level through several mechanisms, such as processing, subcellular localization and decoy. The latter mechanisms call into question lncRNA functions.

Concerning lncRNAs, global genome profiling studies uncovered that the expression of many of them is lymphocyte-specific and change dynamically during T-cell differentiation, suggesting that, also, these functional RNAs may contribute to the development and homeostasis of the immune system [14,15]. This new evidence, together with the observation that cells are able to exchange information not only via hormones, but also via genetic material, is dictating the need to build a model of multilayered control of the development and the differentiation of the immune system cells, as well as of the regulation of the immune response. Perturbations in any component of this regulatory system will be associated with the onset of immune diseases.

This review will explore the proposed multifaceted roles of these classes of ncRNAs in adaptive immunity.

## 2. MicroRNAs

Over the past decades, it has been estimated that only 2% of the mammalian genome encodes for proteins, whereas the vast majority of it is extensively transcribed to give rise to a large transcriptome of ncRNA species [16]. This pervasive transcription, which has been recently unraveled, thanks to the new improved RNA-sequencing technologies, challenges our traditional understanding of RNAs as simple intermediates between genes and proteins. Extensive studies demonstrated that ncRNAs are functional RNA species, involved in the modulation of primary biological functions, and miRNAs, that are, to date, the most characterized class of small ncRNAs, have been described as fundamental to drive the development and differentiation of immune cells [17–20].

**Figure 1:**
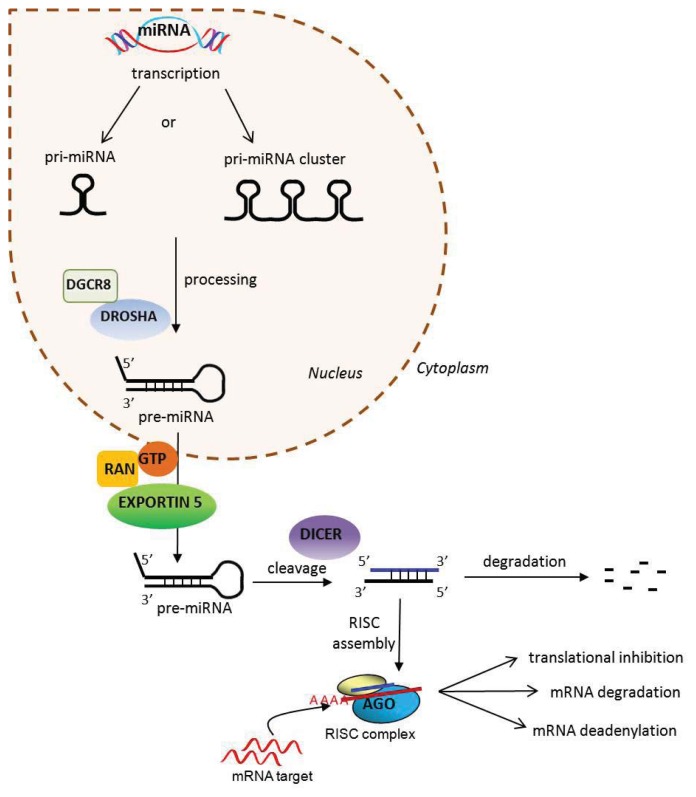
Key steps in microRNA (miRNA) biogenesis and activity. miRNAs originate from the nucleus as pri-miRNA precursor molecules, organized as single transcriptional units or as a cluster of miRNAs, co-transcribed as a polycistronic transcript. They are processed by the RNAse III-type enzyme, Drosha, in association with the RNA-binding protein, DGCR8, into smaller precursor miRNAs (pre-miRNAs), then exported to cytoplasm, where they are cleaved by Dicer to their mature form of 22 nt double stranded miRNA. The guide strand of the mature miRNA is incorporated into the miRNA-induced silencing complex (miRISC), where it binds to target mRNA by partial complementarity with its 3′UTR. This results in translational inhibition, mRNA degradation or mRNA deadenylation of the recognized miRNA target. Ago, Argonaute.

miRNAs are small, single-stranded endogenous ncRNAs, of about 22 nucleotides (nt), many of which are highly conserved through evolution. They are encoded by genes that produce transcripts for either single or multiple miRNAs or by intronic sequences of protein coding genes. Their expression is highly regulated and can be tissue- and time-specific. Following transcription, the primary RNA transcripts (pri-miRNAs), which form a distinctive hairpin structure, are processed by a complex formed by ribonuclease III-type Drosha and DiGeorge syndrome critical region gene 8 (Drosha/DGCR8) and, then, exported from the nucleus into the cytoplasm as pre-miRNA [21]. In the cytoplasm, pre-miRNAs are recognized and cleaved by another endoribonuclease, Dicer, which generates a 22 nt double-stranded miRNA. The double stranded miRNA is unwound, and one strand, the guide strand, is loaded into the RNA-induced silencing complex (RISC). Within the RISC, the mature miRNA interacts with Argonaute (Ago) proteins, driving Ago and other associated factors to partially complementary target sites, mainly located in the 3′ UTR (3′ untranslated region) of the target messenger RNAs (mRNAs) [21–23]. The resulting effect of miRNA-target mRNA interaction inside the RISC complex is the translational inhibition, degradation or deadenylation of the target mRNAs [24,25] (Figure 1).

MiRNAs have been demonstrated to affect the expression of numerous mRNAs within a cell, as well mRNAs can be targets of different miRNAs that act cooperatively. Although the effect of miRNAs on single targets can be relatively modest, the coordinated effect on multiple components of the same pathway adds strength and dynamicity to miRNA-mediated regulation of a biological process.

From the perspective of adaptive immunity, miRNAs have been extensively studied both in physiological and pathological contexts; they have been shown to regulate cell development and differentiation decisions playing a role in lineage commitment and in maintaining lineage stability. These effects are achieved by regulating either, positively or negatively, several signaling pathways.

### 2.1. miRNAs in T-Cell Development

Lymphoid progenitors developed from hematopoietic stem cells in the bone marrow migrate to the thymus to complete their antigen-independent maturation into functional T-cells. Thymic microenvironment directs T-lymphocyte maturation, characterized by the expression of well-defined cell-surface markers: CD4 and CD8, with thymocytes first starting as CD4^−^CD8^−^ double negative (DN), then becoming CD4^+^CD8^+^ double positive (DP) and, lastly, maturing into single positive (SP) CD4^+^ or CD8^+^ T-cells [26].

T-cell-specific deletions of Dicer revealed that the miRNA pathway is required for the normal development of T-cells, as well as for the differentiation of effector T-cell subsets. Indeed, conditional deletions of Dicer in the T-cell lineage resulted in a decrease of T-lymphocytes in thymus and the periphery [8,9,13].

Expression profiling studies, using oligonucleotide arrays (miRNA microarray analysis), small RNA cloning, real-time PCR (polymerase chain reaction) and Next-Generation Sequencing, have shown that miRNA expression changes dynamically during T-cell development and differentiation, implying that the expression of individual miRNAs is associated with different stages of development and different T-cell subsets [27,28]. It was noticed that early B- and T-cell precursors are more closely related to each other than to splenic B-cells and naive T-cells, respectively, and that miRNAs highly expressed in bone marrow progenitor populations were downregulated in thymocytes and other mature lineages. These observations suggested that miRNAs shared among early precursors conceivably regulate their self-renewal and maintenance of an undifferentiated state [29,30]. For instance, one of the most expressed miRNAs in hematopoietic stem cells (HSCs) is miR-125b [29,30], whose levels drop significantly in committed progenitors, indicating that it possibly regulates hematopoiesis at the stem-cell level. This hypothesis is supported by the demonstration that miR-125b increases the size of the hematopoietic stem cell compartment in mice, targeting several component of the pro-apoptotic pathway, as well as genes coding molecules involved in both B- and T-cell differentiation [29,31–33].

The expression of two miRNAs, miR-181a and miR-150, has been shown to be dynamically regulated during thymocyte development.

MiR-181a is transiently upregulated at the late DN to DP stages of T-cell development, while in SP and mature T-cells, its expression is very low [34]. This pattern of expression indicates that miR-181a plays an important role in the development of T-cells in the thymus. Enforced expression of miR-181a in mature T-lymphocytes results in increased sensitivity to peptide antigens, whereas the inhibition of miR-181a in immature DP cells attenuates their sensitivity and impairs both positive and negative selection [35]. miR-181a modulates T-cell receptor (TCR) signaling by targeting several phosphatases that restrain ERK activity. In light of this evidence, it has been proposed that miR-181a may function as an intrinsic antigen-sensitivity “rheostat”, operating during T-cell development, able to quantitatively modulate T-cell sensitivity to antigen, setting a TCR signaling threshold for proper agonist selection [35]. Along this line, expression of miR-181a is altered during autoimmune disease and significantly downregulated in pediatric Systemic Lupus Erythematosus (SLE) patients [36].

Concerning miR-150, it is expressed at high levels in mature naive B- and T-cells and strongly downregulated in their precursors and upon activation. This leads to the conclusion that a common set of miRNAs could be employed in B- and T lineages to regulate similar effector functions, such as tissue homing and cytokine production [37–40]. In particular, miR-150 is strongly upregulated during human T-cell maturation; its expression is low in DN T-cells, increases in DP and CD8^+^ cells and is higher in CD4^+^ cells [20]. miR-150 upregulation in this context could be important to downregulate genes associated with an immature phenotype, such as NOTCH 3, which has been identified as a target of miR-150 [20]. The observation that downregulation of miR-150 confers a growth advantage to transformed lymphocytes corroborated the hypothesis that one of the functions of miR-150 in lymphocyte development is to hamper proliferation during specific stages of maturation to allow for differentiation. Importantly, a proper immune response also requires downregulation of miR-150 after activation of mature B- and T-cells in order to release them from growth arrest and permit rapid proliferation to occur [37].

It is worth mentioning that, besides changes in the expression of specific miRNAs, the dynamic regulation of miRNA expression relies also on precursor processing, which appears to vary during T-cell development. Kirigin *et al*. have reported a significant degree of polymorphisms in mature miRNA ends at the late DN to DP stages, suggesting that a stage-specific processing of miRNA precursors may concurrently affect target recognition, thus contributing to proteome changes associated with T-cell development [29].

### 2.2. miRNAs in T-Cell Function

Antigen-specific naive T-cells, upon activation, proliferate and differentiate into effector T-cells, specialized in either cytotoxic function or cytokine production. Upon antigen recognition, naive CD4^+^ T-cells proliferate and differentiate into distinct types of memory and effector T-cell subsets, that home to different tissue and produce specific types of cytokines. Among CD4^+^ effector T-cells, T-helper 1 (Th1) cells are critical for host defense against intracellular pathogens and secrete interferon-γ (IFN-γ), whereas T-helper 2 (Th2) cells control parasitic infections and secrete interleukin 4 (IL-4), IL-5 and IL-13, important for mucosal barrier function, as well as for the induction of B-cell proliferation. T-helper 17 (Th-17) cells promote the response against extracellular pathogens by producing IL-17 and are also involved in autoimmunity. An additional effector population, more recently described, includes Th9 and Th22 cells, respectively, producing IL-9 and IL-22, whose *in vivo* role has not been clearly elucidated, yet [41]. It has been noted that during T-cell differentiation, the global level of miRNA expression inversely correlates with the activation status of the cells being highest in quiescent naive and memory T-cells and sharply decreasing in proliferating and functionally differentiated effector cells. This occurrence suggests that miRNAs may play a general role in stabilizing the expression of genes involved in the maintenance of a naive state. Accordingly, it has been recently observed that Ago proteins are post-transcriptionally downregulated by ubiquitination upon T-cell activation [42]. Ago proteins’ downregulation, together with miRISC-accelerated turnover, could contribute to a rapid reprogramming of miRNA repertoire in favor of miRNAs whose expression is induced during T-cell activation, such as miR-182 [43].

The human CD4^+^ T lymphocytes-specific miRNA signature changes during differentiation from naive to memory cells. In particular, miR-125b, which is highly expressed in human naive CD4^+^ T-cells and is downregulated during differentiation toward effector T-cells [44], may contribute to maintenance of the naive state of primary human T-cells. In human, miR-125b directly regulates the expression of genes encoding molecules required for the differentiation of different effector T-lymphocytes, such as IFN-γ, peculiar to the Th1 subset, IL-2 receptor-β (IL2RB), which contributes to memory cell identity, IL-10 receptor-α (IL10RA) and the transcriptional repressor, Blimp-1 (PRDM19), which promote T-cell terminal differentiation. Thereby, activation-induced miR-125b down-modulation is associated with increased expression of these genes and acquisition of an effector memory phenotype by CD4^+^ T-cells [44].

A key feature that characterizes the transition from a naive to an activated status of lymphocytes is the active proliferation, triggered by antigen recognition, which leads to the activation of the TCR signaling pathway. Once T-helper (Th) lymphocytes are induced to proliferate, their clonal expansion becomes independent of further antigen stimulation and is fostered by autocrine or paracrine IL-2 production. In this scenario, miR-182, whose expression is induced by IL-2, has been described as an important modulator of Th cell population, by promoting clonal expansion through post-transcriptional inhibition of forkhead box O1 (Foxo1), a suppressor of proliferation expressed in resting Th lymphocytes [42]. This finding is consistent with the present assumption that miRNAs induced during T-cell activation may contribute to downregulating the expression of genes involved in the maintenance of the naive state to allow for effector cell proliferation and differentiation. Moreover, it has been shown that activated proliferating T-cells express mRNAs with shortened 3′ UTR regions and, consequently, fewer miRNA target sites, thus exploiting another mechanism to regulate immune system homeostasis [45].

Among the other miRNAs whose expression is enhanced upon T-cell activation, we will discuss miR-155, miR-146a and cluster miR-17-92 (Table 1).

miR-155, which maps within an exon of the noncoding RNA, Bic [13], has been proven to regulate several aspects of the immune system [62]. miR-155 expression increases upon T-cell activation and controls the differentiation of CD4^+^ T-cells into different Th cell subsets [11,13], as well as the development of regulatory T (Treg)-cells [58,59]. In miR-155 deficient mice, CD4^+^ T-cells preferentially differentiate toward the Th2 subtype. Accordingly, *in vivo* expression data from miR-155-deficient mice and *in vitro* reporter assays indicate that musculoaponeurotic fibrosarcoma oncogene homolog (c-Maf), a potent trans-activator of the IL-4 gene promoter, is a target of miR-155 [13]. Therefore, miR-155 may promote differentiation toward a Th1 phenotype by limiting the expression of IL-4, a cytokine whose expression characterizes the Th2 phenotype.

In addition to its function in innate immunity [12,17,63,64], miR-146a is also known to be implicated in the adaptive immune response. In mice, miR-146a expression is higher in Th1 cells and lower in Th2 cells when compared to naive T-cells, suggesting that miR-146a may be involved in the lineage differentiation of T-lymphocytes [11]. In human T-cells, miR-146a is induced upon TCR stimulation and is highly expressed in central memory T-cells [18]. Consistent with this expression profile, miR-146a has been shown to modulate activation-induced cell death (AICD) by targeting the Fas-associated death domain (FADD) and to impair both activator protein 1 (AP-1) activity and interleukin-2 (IL-2) production induced by TCR engagement [18]. Moreover, the observation of increased miR-146a levels in both synovial tissues and in peripheral blood mononuclear cells (PBMCs) of rheumatoid arthritis patients suggest that miR-146a may promote the survival of self-reactive T-cells in autoimmune diseases [52–55]. Finally, in miR-146a-deficient mice, it has been demonstrated that miR-146a contributes to proper resolution of T-cell response, being a part of the negative feedback loop that modulates TCR signaling to the nuclear factor κ-light chain enhancer of activated B-cells (NF-κB). Indeed, NF-κB, a transcription factor activated upon TCR stimulation, induces the expression of miR-146a, which, in turn, downregulates NF-κB activation through repression of NF-κB activators, TNF receptor associated factor 6 (TRAF6) and interleukin-1 receptor-associated kinase 1 (IRAK1) [12,64]. NF-κB binds to the human miR-17-92 promoter, indicating that, also, the miR-17-92 cluster may be one of the intracellular signaling components that promotes proliferation and effector differentiation in response to antigen stimulation. miR-17-92 expression is induced upon T-cell activation during viral infection, while it is downregulated after clonal expansion and during memory cell differentiation [56]. Notably, failure in miR-17-92 downregulation leads to defective central memory cell development [56]. In mice, ectopic expression of the miR-17-92 cluster in the lymphocyte compartment induces a severe lymphoproliferative disease characterized by an expansion of almost all lymphocyte populations, especially the CD4^+^ T subset [46]. Moreover, B- and T-lymphocytes from transgenic mice show increased proliferation and survival after activation *in vitro* [46]. The phenotype associated with miR17-92 cluster overexpression can be, at least in part, the result of the downregulation of two direct targets of the miR17-92 cluster: bcl-2 interacting protein (Bim), a pro-apoptotic protein belonging to the Bcl2 family, and the tumor suppressor phosphatase and tensin homolog (PTEN).

Studies comparing naive, effector and memory CD8^+^ T-cells show that a small set of miRNAs is downregulated in effector T-cells compared to naive cells, but also that their expression tends to come back in memory T-cells [52]. Activated T-cells committed to a central memory fate activate a program that enables the acquisition of a central memory phenotype through downregulation of miR-155 and upregulation of miR-150, whose expression is suppressed upon T-cell activation (Table 1). This opposite regulation of miR-150 and miR-155 with respect to the activation state appears coherent, as an effort of the emerging central memory T-cells to distance themselves from the activated state and acquire a maintenance state [39].

### 2.3. miRNAs in Treg Cells

Treg cells produce inhibitory cytokines, such as IL-10 and transforming growth factor-β (TGF-β), which help to limit immune responses and prevent autoimmunity by suppressing T-lymphocyte activity. Treg cells are critical for the maintenance of immune cell homeostasis, as evidenced by the lethal condition consequent to their ablation in mice [65]. miRNAs have been established to be essential for Treg cell function: indeed, conditional deletion of Drosha or Dicer in forkhead box protein P3^+^ (Foxp3^+^) Treg cells induces a fatal, early-onset autoimmune pathology indistinguishable from that observed in Foxp3-knockout mice devoid of Treg cells [10,66,67]. The loss of suppressor function observed in Dicer-deficient Treg cells is most likely due to the deficiency of miRNAs that are over-represented in these cells. Among them, miR-10a, miR-155 and miR-146a have been shown to contribute to distinct aspects of Treg cells homeostasis and function (Table 1). miRNAs confer robustness to differentiation processes by tuning gene expression networks; therefore, they may help to preserve certain phenotypes by targeting transcriptional factor pathways that promote alternative fates. An interesting recent study [57] has shown that miR-10a contributes to the stability of the Treg phenotype and limits the conversion of Treg cells into T-follicular helper (TFH) by targeting the transcriptional repressor B-cell leukemia/lymphoma 6 (Bcl-6). miR-10a is expressed at high levels in naturally occurring Treg (nTreg) cells and is induced by TGF-β and retinoic acid (RA) during inducible Treg (iTreg) cell differentiation from CD4^+^ T-cells. Actually, TGF-β induces retinoic acid receptor α (RARα) expression, which, upon stimulation by RA, activates miR-10a expression. miR-10a directly targets not only Bcl-6, a master positive regulator of TFH differentiation, but also nuclear receptor corepressor 2 (Ncor2), a corepressor of RARα and, in so doing, activates a positive feedback loop that amplifies its own induction by RA. On the other hand, the miR-10a-induced downregulation of Bcl-6 [57], a negative regulator of T-cell-specific T-box transcription factor (Tbet), elicits higher levels of Tbet, which is known to inhibit Th17 differentiation. Therefore, miR-10a modulates T helper cell stability by restraining, either directly or indirectly, multiple differentiation pathways.

miR-155 not only favors T-cell differentiation toward a Th1 phenotype, but also influences Treg cell homeostasis. miR-155-deficient mice have reduced numbers of Treg cells, both in the thymus and periphery; however, miR-155-deficient Treg cells maintain their suppressor activity [60]. Remarkably, Foxp3, the key transcription factor controlling Treg cell development and function, upregulates the expression of miR-155, which, in turn, positively regulates Treg cell differentiation by targeting the suppressor of cytokine signaling 1 (Socs1) [60]. Accordingly, T-cell-specific deletion of Socs1 results in an increase in the proportion and absolute numbers of Treg cells in the thymus without affecting their suppressive function [59,60,68]. Therefore, miR-155 stabilizes the transcriptional program induced by Foxp3 by repressing genes whose expression counteracts Treg cell differentiation. In contrast to miR-155-deficient mice that exhibited a decreased number of Treg cells in thymus and in the periphery, miR-146a-deficient mice showed an increased number of Treg cells in the periphery; however, miR-146a-deficient Treg cell suppressor activity was seriously compromised [61]. Fatal immune-mediated lesions observed in mice in the presence of miR-146a-deficient Treg cells were accompanied by sharply augmented Th1 responses and were dependent upon increased amounts of IFNγ. Li-Fan Lu *et al*. demonstrated that miR-146a ensures Treg cell-mediated regulation of Th1 responses, in a significant way, through targeting signal transducer and activator of transcription (STAT1), a transcription factor that regulates IFNγ expression [61].

### 2.4. miRNAs in B-Cells

In the adaptive immune response, B-cells are responsible for antibody-mediated response. B-cell development begins in primary lymphoid tissue, with subsequent functional maturation in secondary lymphoid tissue. It is a continuum of stages defined by the expression and re-arrangement of functional B-cell receptor (BCR)/immunoglobulin (Ig) genes [2]. The first developmental stage exhibiting commitment to the B-cell lineage is called pro-B and is characterized by rearrangement of the Ig heavy chain. In the pro-B stage, Iga (CD79a) and Igβ (CD79b) are expressed at the cell surface. Rearrangements at the immunoglobulin locus result in the generation and surface expression of the pre-B-cell-receptor (pre-BCR), composed of an Igμ heavy chain and surrogate light chains and, finally, a mature BCR, capable of binding antigen [2]. Mature B-cells that move into the periphery can be activated by antigen, undergo clonal expansion and become an antibody-secreting plasma cell or a memory B-cell, which will respond more quickly to a second exposure to antigen. Unlike conventional B-cells (B-2 cells), which originate from adult bone marrow progenitors, another population of B-lymphocytes, called B-1, appear during fetal life and express surface IgM, but little or no IgD. In mouse, B1 cells can be further subdivided into B-1a (CD5^+^) and B-1b (CD5^−^) subtypes [69]. B1 lymphocytes do not undergo much class switch, thus producing antibodies with low affinity and having been implicated in the pathogenesis of autoimmune diseases and many chronic leukemias.

Conditional ablation of Dicer or Ago2 in early B-cell progenitors results in an almost complete block of B-cell development at the pro-B to pre-B transition, which underscores the relevance of miRNA function in B-cell development [70]. Moreover, Dicer-deficient B-cells show increased expression of terminal deoxynucleotidyl transferase (TDT) and increased antibody diversification [47,70,71]. Several specific miRNAs have been proven to control B-cell development, which is impaired in Dicer-deficient mice (Table 1). The miR-17-92 cluster is highly expressed in progenitor B-cells, and its expression decreases during B-cell maturation, suggesting that it is a positive regulator of B-cell differentiation [47]. In miR-17-92-deficient mice, transition from pro-B-cells to pre-B-cells is compromised, and increased apoptosis occurs, which correlates with higher expression of the pro-apoptotic protein, Bim, a target of the miR-17-92 cluster [46]. Accordingly, overexpression of the miR-17-92 cluster, often detected in human B-cell lymphomas, possibly facilitates transformation by inappropriate repression of the same pro-apoptotic gene, Bim, and the tumor suppressor, PTEN [56]. Another positive regulator of B-cell differentiation is miR-181a, whose ectopic expression in hematopoietic stem cells leads to an increased production of B-lineage cells [72]. Conversely, the overexpression of miR-150 in hematopoietic progenitor/stem cells significantly reduces the mature B-cell population by targeting c-Myb, a transcription factor critically important in B-cell development. MiR-150 is selectively expressed in mature, resting B- and T-cells, but not in their progenitors [9,38]. The expression pattern of c-Myb in B-cells inversely correlates with that of miR-150: it is highly expressed in progenitor cells and downregulated in mature B-lymphocytes. Premature downregulation of c-Myb by ectopic miR-150 expression triggers apoptosis during the pro-B stage, determining the observed phenotype. These findings substantiate the view that higher levels of miR-150 are required during the pre-B to mature B-cell transition to downregulate c-Myb expression and guarantee normal B-cell development [37,38].

The constitutive expression of miR-34a, whose expression is downregulated at the pro-B to pre-B transition, determines a block of the early B-cell development with an accumulation of pro-B-cells and a reduction in pre-B-cells and mature B-cells [48]. These effects have been proven to be the result of targeting Foxp1, a transcription factor required for early B-cell development. Interestingly, the investigators highlight that the effect of miR-34a on the B-cell developmental pathway [48] is consistent with previously reported abnormalities associated with a deficiency of p53, namely, an increased number of pre-B-cells, as well as mature B-cells, with the latter finding being also a consequence of the loss of miR-34a function. This evidence implies a connection between p53 and Foxp1 through the action of miR-34a, which has been demonstrated to be a direct transcriptional target of p53 and to participate in the control of inappropriate cell proliferation [73,74].

miRNAs also regulate B-cell development in the periphery (Table 1). Indeed, Dicer ablation in mature B-cells results in augmented B-cells in the marginal zone and diminished follicular B-cells. miR-155, whose levels are low in progenitor and mature B-cells and are induced upon B-cell receptor ligation, has been implicated in the differentiation of normal activated B-cells. miR-155-deficient mice show multiple defects in the germinal center (GC) response, such as a decreased number of GC B-cells, decreased IgG production, and decreased affinity maturation. Using mouse models, miR-155 has been demonstrated to affect the regulation of the GC response through modulation of cytokine production [13,49] and by direct post-transcriptional regulation of the transcription factor, Pu.1 [75], and activation-induced cytidine deaminase (AID) [50,76].

### 2.5. miRNAs and the Anti-Viral Immune Response

The ability of small noncoding RNAs to act as immune sentinels was initially described in plants, nematodes, fungi and insects [22,55,64,77] in which the RNAi-mediated recognition of viral RNAs has evolved as a defense mechanism against RNA viruses. In mammals, another class of small RNA, the piwi RNAs (piRNAs), has been shown to suppress transposable element (TE) expression and mobilization through their silencing [78]. Recently, human cellular miRNAs have also been described as intracellular immune mediators, adopted by the organism to fight viruses. The short seed sequence and the tolerance for mismatches represent peculiar properties of miRNAs, which confer them the capability to recognize and downregulate viral mRNAs, required for successful replication and host infection. In mammals, the repression of Dicer and, hence, miRNAs production, confers increased susceptibility to human immunodeficiency virus type 1 (HIV-1), influenza A virus and vesicular stomatitis virus (VSV) [79–81]. In particular, the anti-HIV effects of Dicer are mediated by two cellular miRNAs, miR-17-5p and miR-20a, which target histone acetylase p300/CBP-associated factor (PCAF), an important cofactor of the HIV protein, Tat [79]. Two other miRNAs, miR-93 and miR-24, instead, contribute to the host defense against the rhabdovirus vesicular stomatitis virus (VSV), by targeting complementary sites in the VSV genome [81]]. On the other hand, viruses evolved several mechanisms to evade host defense, including quenching of the miRNA machinery [79], and, also, co-opted miRNA genes to suppress the host antiviral response; therefore, further studies are required to fully understand the role of miRNAs in the complex interaction between viruses and their mammalian hosts.

### 2.6. miRNAs and Autoimmunity

Given the pivotal role of miRNAs in the modulation of development, maturation and function of lymphocytes in normal immune response, it is not surprising that they have been also involved in the pathogenesis of several immune disorders. An integral aspect of miRNA-mediated regulation is their ability to function over a narrow range of concentration during normal immune responses; they induce subtle variations in the expression of key proteins that, if inappropriate, may lead to an overt pathological phenotype. Many immune diseases arise from the altered balance of gene expression patterns rather than from heavy changes at the levels of protein-coding regions of the genome. As in the case of multiple polymorphisms, which individually have a small effect [82], miRNAs exert their regulatory role through the subtle individual modulation of multiple targets involved in a common signaling pathway rather than through a strong repression of unique targets, thus contributing to lowering the threshold for the onset of an autoimmune response. Notably, up to one half of immune genes are predicted to be under the direct regulation of miRNAs; therefore, mutations that directly affect miRNA expression or alter the miRNA biogenesis pathway may have a causative effect during disease initiation and progression, owing to inappropriate repression or de-repression of key protein targets.

The tight regulation of immune cell activation and the proper selection of a functional, non-self-reactive repertoire of specific antigen receptors is at the base of the fine balance between a normal immune response and the development of autoimmunity. Because of the miRNA ability to regulate the survival and death of lymphocytes [18,51], control over their expression is essential to prevent adaptive immune cells from dysregulated proliferation and activity leading to leukemia and autoimmunity [83,84]. Several miRNAs exhibit a different expression profile in patients affected by autoimmune diseases compared to normal subjects [84–86], suggesting a causative role for the pathogenesis of human autoimmune diseases, including rheumatoid arthritis (RA), psoriasis and SLE. The expression of some miRNAs, like miR-146a and miR-155, appears to be constantly dysregulated in the aforementioned autoimmune diseases.

SLE is an autoimmune disease characterized by the loss of immune tolerance, resulting in activation and expansion of autoreactive CD4^+^ T-helper cells [87]. In MRL/lpr mice, a murine model of SLE characterized by the accumulation of CD4^+^CD25^+^Foxp3^+^ Treg cells in lymphoid organs [88], the onset of the autoimmune disease correlates with a reduced functional capacity of Treg cells that show a distinct phenotype (*i.e.*, increased CD69 and reduced CD62L expression). CD62L, an l-selectin, which determines the homing properties of T-lymphocytes, is a target of miR-155, one of the miRNAs upregulated in SLE. These data indicate that miR-155 dysregulated expression may directly contribute to the accumulation of Treg cells in the lymphoid organs of MRL/lpr mice [89]. On the contrary, miR-146a expression is reduced in the PBMCs of SLE patients. MiR-146a downregulation inversely correlates with clinical disease activity and with interferon (IFN) scores and may be responsible for the IFN overproduction observed in SLE. This phenotype is consistent with miR-146a-mediated repression of the type I IFN pathway, through the direct targeting of STAT-1 and IFN regulatory factor 5 (IRF5) [90].

In RA patients, upregulation of miR-146a and miR-155 has been found in PBMCs, as well as in the synovial tissue [52–54]. miR-146a also is highly expressed in CD4^+^ T lymphocytes from RA patients and exhibits a strong correlation with increased levels of TNFα [86]. Interestingly, T-cells isolated from joint tissue and synovial fluid of RA patients show an activated and memory phenotype and are resistant to apoptosis, although the high levels of pro-apoptotic factors, like Fas-ligand (FasL), TNFα and TNF-related apoptosis-inducing ligand (TRAIL). Given that our study [18] and others [54,91] demonstrated an anti-apoptotic role of miR-146a in T-lymphocytes and other cell types, the increased survival of T-lymphocytes in RA patients could be in part explained by the enhanced expression of miR-146a. Another anti-apoptotic miRNA that has been related to the pathogenesis of autoimmune disorders is miR-17-92. Its overexpression results in a decreased activation-induced cell death, sustained lymphoproliferation and a marked presence of serum autoantibodies and lymphoid infiltrates [46]. Although the development of T-lymphocytes does appear normal, the relevant increase in the number of mature CD4^+^ T-cells and their activated profile strongly suggest a failure of peripheral tolerance. The molecular mechanisms contributing to this immune disease-related phenotype of miR-17-92 transgenic mice are based on the downregulation of the tumor suppressor, PTEN, and the pro-apoptotic, Bim [46]. An interesting study demonstrating the involvement of miRNAs in the possible alteration of autoimmune-related signaling pathways was performed in 2007 in sanroque mice, a murine model resembling a lupus-like autoimmune syndrome [92]. These mice are homozygotes for an M199R mutation in the ROQ domain of Roquin, a protein that binds to mRNAs and targets gene expression post-transcriptionally. The evident accumulation of autoimmune lymphocytes in this murine model depends, at least in part, on the failure to repress the expression of the co-stimulatory molecule inducible co-stimulatory molecule (ICOS), particularly in naive T-cells. Roquin normally limits ICOS expression by promoting the degradation of ICOS mRNA, by a mechanism that involves the recognition of a conserved miRNA binding sequence within the 3′ UTR. It has been reported that miR-101 is required for the Roquin-mediated degradation of ICOS mRNA [92], and the alteration of the seed site for miR-101 in the 3′ UTR of ICOS disrupts the inhibitory activity by Roquin. These results demonstrate a critical miRNA-mediated regulatory pathway that prevents lymphocyte accumulation and autoimmunity. Further evidence supporting a role of miRNAs in autoimmunity has been provided by Ebert *et al*., who demonstrated the relevance of miR-181a expression for the elimination of self-reactive thymocytes; indeed, the inhibition of miR-181a expression during thymic development converts endogenous positively selecting peptides into autoantigens [93].

Along with the miRNA-mediated deregulation of disease susceptibility genes, few studies also detected autoantibodies targeting key components of the RNAi/miRNA machinery, including Argonaute proteins (referred to as anti-Su antibodies) and Dicer, in patient blood. The anti-Su autoimmune sera recognize several members of the Ago protein family, which are known to associate into RISC and which have a high degree of sequence identity. Despite that the first characterization of autoantigens recognized by anti-Su autoantibodies [94] had been provided in 1984, the disease specificity of anti-Su antibodies has been addressed in only a few studies, so far. They were initially reported to be specifically associated with SLE [94], but later studies showed that anti-Su antibodies are, instead, found in a variety of systemic rheumatic diseases [95]. The existence of an autoimmune response directed against macromolecular complexes involved in the post-transcriptional regulation of gene expression may indicate the involvement of the miRNA biogenesis pathway in the production of autoantibodies, further supporting the intriguing hypothesis that dysfunction of the RNAi/miRNA machinery lies at the core of the pathogenesis of autoimmune diseases.

## 3. Long Noncoding RNAs

At least 80% of mammalian genome transcription results in the generation of lncRNAs. It has been estimated that 10,000 to 200,000 different types of lncRNAs are transcribed in the human genome, but functional and molecular mechanisms have been elucidated only for a few [96]. Given their unexpected abundance, lncRNAs were initially considered as spurious transcriptional noise, without biological function, and this assumption was corroborated by the observation that many of the transcripts are expressed at very low levels and exhibit no sequence conservation [97,98]. Nevertheless, it is now apparent that lncRNAs are functional, and many of them are differentially expressed in particular developmental stages.

**Figure 2:**
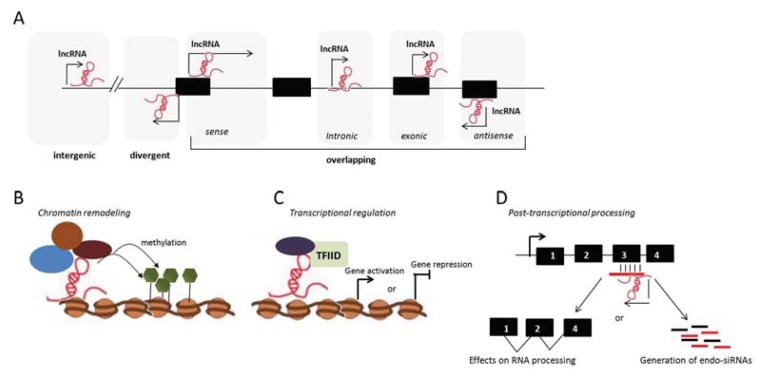
Long non coding RNAS (lncRNAs): mechanism of action and genomic organization. Illustrative representation of lncRNAs classification relative to their genomic position or mechanism of action. (**A**) lncRNAs are generally classified according to their proximity to protein coding genes in the genome: intergenic lncRNAs are distant from protein coding regions; divergent lncRNAs are located on the opposite strand closed by the transcription starting site of a protein coding gene; intragenic lncRNAs overlap protein coding genes and can be sense, antisense, intronic or exonic; (**B**) lncRNAs can recruit chromatin remodeling complexes to specific genomic loci, to epigenetically mark the region for gene silencing; (**C**) lncRNAs can permit the recruitment of transcription factors activating gene expression or can interfere with the transcription machinery, occluding the access of transcription factors and, thereby, silencing the gene; (**D**) lncRNAs that are antisense to protein coding genes may regulate the splicing or induce the degradation of their corresponding mRNA transcripts.

LncRNAs comprise many different transcripts, ranging from several hundreds to ten-thousand nucleotides and can be classified according to their genomic position as intergenic or intragenic lncRNAs (Figure 2A). Intergenic lncRNAs are located distant from protein coding regions, whereas divergent lncRNAs, often referred to as bidirectional lncRNAs, are located on the opposite strand from a coding gene whose transcription is initiated in close proximity. Intragenic (overlapping) lncRNAs are transcribed along one or multiple exons (exonic lncRNA) or introns (intronic lncRNA) of another transcript on the same or opposite strand and can then be spliced into a mature RNA (Figure 2A).

Unlike miRNAs, lncRNAs possess heterogeneous molecular mechanisms of action, making them master regulators of intracellular networks and pathways in both physiology and diseases [96]. Some mammalian lncRNAs have two unique relevant properties. The first one is the ability to target a single location through the use of a large sequence space. While transcription factors are effectively recruited to the DNA promoter region by recognizing short DNA motifs, which typically occur thousands of times in the genome, lncRNAs, like Tsix and RepA/Xist, are able to deliver epigenetic complexes to a unique site, thus providing a spatio-temporal regulatory specificity not achievable with proteins and small RNAs [63,99]. A second characteristic of some lncRNA-mediated regulation is the “allelic memory”. Proteins loose the memory of their transcriptional origin when the mRNA is shuttled to the cytoplasm to be translated; lncRNAs, instead, remain tethered to the site of transcription and can, therefore, have allele-specific action. Differently from miRNAs, which are mainly post-transcriptional repressors, lncRNAs have a full range of functions, being able to act as scaffold RNAs, co-activators or co-repressors of trans-acting RNAs and epigenetic regulators of protein-coding gene expression. In particular, they can mediate epigenetic changes by recruiting chromatin remodeling complexes to specific genomic loci, thus altering the chromatin status at gene promoters to affect gene expression [100] (Figure 2B). Another major mechanism of lncRNA action is transcriptional regulation, exerted through specific interaction with transcription factors or other protein components of the transcriptional machinery. LncRNAs may sequester transcription factors in the cytoplasm, preventing their translocation into the nucleus [101] or occlude a transcription factor binding site, thereby promoting gene repression [102]. LncRNAs can also promote gene expression, through interactions with components of the core transcriptional machinery, thus enhancing the specific binding of transcription factors to promoter regions (Figure 2C). Moreover, lncRNAs have been implicated in posttranscriptional regulation, either by the direct regulation of alternative splicing through base-pairing interactions or by gene silencing via the generation of endo-siRNAs and the consequent degradation of the targeted transcript (Figure 2D).

### 3.1. lncRNAs and the Adaptive Immune Response

In contrast to the wealth of recent evidence demonstrating lncRNA involvement in cancer, at present, a role of lncRNAs in immune system regulation is attested by only a handful of mechanistically distinct examples. The importance of lncRNAs in the immune system has been recently highlighted by a report that identified and characterized a set of lncRNAs preferentially expressed in naive and memory mammalian CD8^+^ T-lymphocytes [14,92]. Over 1000 lncRNAs have been detected and characterized in humans and mice, many of which expressed in a stage- or cell-specific manner. In particular, the authors identified 96 lymphoid-specific lncRNAs. Interestingly, 29 of these transcripts were specific for CD8^+^ T lymphocytes, 21 lncRNAs were significantly modulated during the T-cell differentiation process and 81 were regulated during effector T-cell activation [14]. These expression patterns strongly suggest a possible role of lncRNA in the modulation of the key cellular processes of T-lymphocyte biology. Remarkably, lncRNAs, as well as the recently discovered large intergenic noncoding RNAs (lincRNAs), have been shown to regulate the expression of adjacent protein-coding genes. Actually, several of the lncRNAs identified in the mentioned study are associated with protein-coding genes that exert key functional roles in T-cells. For example, an lncRNA, AK009498, overlaps the transcription start site of caspase-8 (FLICE)-like inhibitory protein (Flip), a gene fundamental for the survival and development of T-lymphocytes. Flip is upregulated during the transition to an activated or a memory phenotype, whereas the host lncRNA is downregulated, thus suggesting a potential negative function of AK009498 [14] (Table 2).

Nettoie Theiler’s Pas Salmonella (NeST), also called Theiler’s Murine Encephalitis Virus Possible Gene1 (TMEVPG1), a nuclear RNA initially described as involved in the control of viral load during persistent infection, is the first lncRNA of the immune system that has been proven to regulate the expression of a master cytokine, such as IFN-γ [103]. This lncRNA is expressed selectively in Th1 cells relative to Th2 and Th17 cells, and its expression depends on Th1-specific transcription factors, STAT4 and Tbet [104]. Moreover, NeST is located adjacent to the IFNγ locus in both humans and mice and is encoded in antisense respect to the IFNγ gene (Table 2). Recent evidence demonstrated that NeST acts in trans and positively regulates the IFNγ gene at the transcriptional level, by interacting with chromatin-modifying complexes. More precisely, it binds WDR5, a member of the histone H3 lysine 4 methyltransferase complex, and promotes permissive methylation marks on histone H3 at the IFNγ locus. The effect of such epigenetic control is the modulation of the host response to viral and bacterial pathogens, thus suggesting a role for lncRNAs as functional links between immune regulation and susceptibility to infections [105]. Indeed, alteration of NeST expression could contribute to differences in T-lymphocyte response and disease susceptibility. The discovery of functional intergenic lncRNAs, whose expression is critical for proper gene regulation, may explain the relevance of some intergenic regions as heritable causes of human diseases.

Another interesting feature of lncRNAs is that a small number of lncRNA genes host in their sequence small RNAs and may be processed into, and exert their effects via, smaller functional ncRNAs. Pang *et al*. identified 18 lncRNAs expressed in murine CD8^+^ T-lymphocytes overlapping with annotated miRNAs and 21 that harbor internally small nucleolar RNAs (snoRNAs) [14]. In this context, AK053349, one of the most highly upregulated lncRNA during effector T-cell activation, partially overlaps miR-17-92, a cluster of miRNAs, often amplified in lymphomas, whose enforced expression in mouse lymphocytes results in lymphoproliferative disease and autoimmunity [46]. Furthermore, miR-142-5p and miR-142-3p, which have been reported as highly expressed in naive T-cells and are involved in the modulation of Foxp3 in Treg lymphocytes, are hosted in the first intron of a lncRNA (AK020764), strongly upregulated in CD8^+^ T-cells [14] (Table 2). The colocalization could imply a functional link, but it has not yet been verified.

Finally, lncRNAs may influence cell regulatory networks not only at the transcriptional level, but also by controlling protein trafficking, as it has been shown for the noncoding repressor of NFAT (NRON), a negative regulator of the transcription nuclear factor of activated T-cells (NFAT). NRON is an lncRNA enriched in lymphoid tissues, such as the thymus, the spleen and the lymph nodes. This lncRNA has a cytosolic localization and has been proven to interfere with NFAT function by interacting with several proteins (Table 2). One of these proteins is importin beta1 (KPNB1), which, in the presence of NRON, sequesters NFAT in the cytosol. In addition, the interactions of NRON with two other proteins, the serine/threonine protein, phosphatase 2A (PP2A), and the IQ motif containing GTPase activating protein 1 (IQGAP1), may also be significant [101]. These examples underscore the possibility of limitless mechanisms of gene regulation based on cooperation between ncRNAs and proteins.

### 3.2. lncRNAs as Biomarkers in Autoimmunity and Leukemia

Genome-wide analysis identified a number of highly conserved transcripts, some of which do not encode for proteins and are, therefore, considered non-genic. Among these, the ultra-conserved regions (UCRs) are noncoding genomic sequences of over 200 bases, located in both intra- and inter-genic regions, and are strongly conserved between human, mouse and rat genomes. The unexpected degree of conservation of UCRs among distant species suggests that they may have essential functional importance for the ontogeny and phylogeny of mammals and vertebrates, in general. A large proportion of transcripts derived from these UCRs have significant RNA secondary structures and are components of clusters containing other sequences with functional noncoding significance [106]. Notably, the highest fraction of transcribed UCRs was found in B-lymphocytes, and distinct UCR signatures have been described as associated with human leukemias and carcinomas. In particular, Calin *et al*. reported a differential expression of 19 UCRs (eight up- and 11 down-regulated) in chronic lymphatic leukemia (CLL) with respect to normal hematopoietic tissues [106,107]. The authors also identified a cluster of seven UCRs (uc. 347 to uc. 353) located within the cancer-associated genomic region (CAGR), and two of them—uc. 349A(P) and uc. 352(N)—are among the transcribed UCRs (T-UCRs) differentially expressed between normal and malignant B-CLL CD5^+^ cells [106]. Furthermore, the authors correlated a signature of five T-UCRs, namely, uc. 269A(N), uc. 160(N), uc. 215(N), uc. 346A(P) and uc. 348(N), with different CLL prognosis groups, consistent with the expression of 70-kDa zeta-associated protein (ZAP-70), an established prognostic marker for CLL. These data suggest that T-UCRs could be candidate genes for cancer susceptibility. Another interesting aspect emerging from this study is the finding that the expression levels of the five T-UCRs described above negatively correlated with a miRNA expression signature reported in CLL [107]. This evidence represents just an example of how lncRNAs and small RNA biology are strictly connected: a significant fraction of lncRNAs seem to act as precursors for small RNA species, in particular for miRNAs. These findings raise the possibility of complex regulatory mechanisms between lncRNAs and small RNAs, suggesting a possible interplay between the different classes of noncoding RNAs.

Given the emerging role of lncRNAs in biomolecular regulatory interactions within cells and their relevance in the etiology of human disease and cancer, it becomes highly pertinent and imperative to perform an exhaustive evaluation of their functionalities in order to shed light on the specific pathways and regulatory circuits in which they are involved.

## 4. Secreted Noncoding RNAs

The introduction of Next-Generation Sequencing (NGS) techniques led to the identification of a large number of unexpected ncRNAs, not only at the level of different human tissues, but also in extracellular fluids (e.g., serum, plasma, urine, milk and saliva) [108–112]. The study of circulating RNA populations has almost exclusively focused on miRNAs, most likely due to the availability of array hybridization techniques to detect these small RNAs and, also, because of their recognized role in several biological processes. Alterations in the level and composition of these extracellular miRNA populations are strictly related to different pathologies, including cancer [20,108], diabetes [108] and tissue injury [113]. Extracellular ncRNAs circulate in human plasma within non-vesicular Ago2 ribonucleoprotein complexes, which confer them stability [114]. Alternatively, they can be stably carried in body fluids, as packaged within membranous vesicles (including exosomes, shedding vesicles and apoptotic bodies) [115–117] and spread signals that affect neighboring or distant cells. These different types of microvesicles protect ncRNAs from degradation during systemic transport and, also, are responsible for the specific delivery of them to recipient cells. Exosomes are small vesicles ranging from 30 to 100 nm in size, originated from endosomes. They consist of a lipid bilayer membrane surrounding a small amount of cytosol. The formation and release of exosomes are tightly regulated by multiple signaling mechanisms. Different stimuli can influence exosome secretion, including bacterial lipopolysaccharide on macrophages and dendritic cells [118]. Shedding vesicles are much larger than exosomes and are heterogeneous in size, ranging from 100 nm to 1 μm [119]. The presence of a ncRNA pool has been reported in exosomes and shedding vesicles derived from a variety of sources, including mast cells [115] blood [120], stem cells [121] and adipocytes [122]. The existence of different secretion mechanisms suggests that specific ncRNA populations may be delivered from different cell types and, therefore, have different fates and functions. It has been speculated that differences between vesicle-enclosed and Ago2-associated ncRNAs may reflect cell type-specific ncRNA release mechanisms. Additional studies are needed to unravel the pathways responsible for ncRNA loading and secretion and to better define the biological function of the different circulating ncRNA species.

### Role of Secreted ncRNAs in Immunological Processes

The first indication of the horizontal transfer of nuclei acids in mammalian cells reported the release of miRNAs and mRNAs by mast cells and their functional transfer to vesicle-targeted cells [115]. This evidence, together with intensive studies describing the delivery of ncRNA-loaded exosomes by immune cells [123,124], strongly suggests that secreted RNAs could represent a new intricate level of cellular communication and regulation of immunological processes. To mount an effective immune response, different immune cells need to exchange information and develop highly specialized structures, called “cell synapses”. During the formation of immunological synapse (IS) between T-lymphocytes and antigen presenting cells (APCs), membrane and transmembrane-associated molecules are rearranged into a highly organized structure at the T-cell-APC contact site [125]. The formation of IS is consequent to antigen recognition and allows the initiation and tuning of T-lymphocyte activation. Interestingly, it has been reported that miRNAs are exchanged during cognate immune interactions, and this delivery is strictly dependent on the formation of IS [124]. These specialized structures promote the unidirectional transfer of miRNA-loaded exosomes from T-lymphocytes to APCs, and this kind of genetic communication is antigen-driven and CD63-dependent. Synaptically transferred miRNAs are functional in recipient cells, as demonstrated by Mittelburn *et al*. [124], showing that antigen-dependent transfer of miR-335 from T-lymphocytes to APC resulted in the targeting of specific genes, involved in the modulation of immune response. Notably, not all miRNAs can be incorporated into exosomes, and their presence into delivered microvesicles is a selective process. The miRNA composition in circulating RNAs does not merely reflect the cellular miRNA pool: certain miRNAs are equally abundant between cellular and extracellular environment; others (like miR-760, miR-362, miR-654-5p and miR-671-5p) are significantly more abundant in exosomal fractions derived from all cell types; and finally, another set (for example, miR-21-3p, miR-101 and miR-32) are, instead, more represented in cells than in exosomes [124]. Moreover, it has been also observed that there is a higher similarity between miRNAs composition in exosomes of different cellular origin than between shuttle RNA and their corresponding cellular miRNAs [123,124]. These data demonstrate that specific miRNA populations are selectively sorted into shuttle RNA, strongly suggesting that cells specifically release these RNA molecules to affect the function of target cells. However, to date, the precise mechanism controlling this selection is still unclear. Together with synaptic-dependent shuttling of miRNA from T-cells to APC, another important source of RNA-loaded exosomes is represented by dendritic cells (DCs), which have a role in the activation of T-lymphocytes. In that context, among the most representative miRNAs vehiculated by exosomes, miR-223 and miR-93 have validated target genes that play important roles in immunity. In particular, miR-223 downregulates myocyte enhancer factor 2C (MEF2C), involved in the transcription of interleukins, whereas miR-93 targets STAT3, thus affecting T-cell activation processes [123]. More interestingly, profiling of shuttle RNA isolated from DC-derived exosomes also revealed the presence of many small noncoding RNA species, other than miRNAs, that could act as regulatory RNAs, in particular, snoRNAs. Altogether, this first piece of evidence suggests a wide range of biological effects that could be mediated by shuttle RNA, and the recent findings that the shuttle RNA population is not restricted to miRNAs, but includes other ncRNAs reciprocally exchanged between immune cells, provide a further level of cellular inter-communication that contributes to affecting the activation and the efficacy of immunological response.

## 5. Concluding Remarks

The proper functioning of the immune system is accomplished through several steps of development and differentiation that must be strictly regulated to respond, at the right time and with the appropriate effector cells, to different insults. Thanks to the advancement of technologies that allow us to have a global picture of which proteins and functional RNAs are expressed in a cell at a defined time, we have now the instruments to dissect the complexity of this regulation, which is time- and cell-specific, allowing the immune system to dynamically adapt to the many challenges it faces. The crosstalk between signaling pathways and epigenetic processes during development and cell differentiation is still poorly understood. Changes in these networks might lead to deregulation of gene expression, which ultimately results in diseases, e.g., autoimmunity and cancer.

It is conceivable that transcriptional control, mediated by epigenetic modifications, is preferentially responsible for the developmental decision, while posttranscriptional control plays a major role during activation and differentiation. This concept is particularly relevant in the immunology field, where recent advances in the “-omics” techniques have revealed a multilayered network composed of protein-coding RNAs, as well as of ncRNAs.

Although the miRNA and lncRNA pathways were initially believed to be independent and distinct, the lines distinguishing them continue to fade. There is now compelling evidence that, despite their differences, these distinct ncRNA pathways are interconnected, interact, compete and rely on each other at several levels as they regulate genes and protect the genome from external and internal threats. However, there are still major gaps in our understanding of their function at the molecular level. It is becoming evident that miRNAs, more than being molecules that act at the post-transcriptional level to switch on and off the expression of specific genes at a precise stage, have a more intricate role. On the one hand, being part of regulatory loops, miRNAs may contribute to the maintenance of a certain state of the cell; on the other hand, because of their transient rising, induced by external stimuli and activation of specific signaling pathways, miRNAs help to rapidly remodel the proteome of a cell, rendering it functional for the needs of the organism. Furthermore, we have now to consider the primary contribution of lncRNAs, regulatory molecules that have the prerogative to join some functional peculiarity of proteins and the capability to recognize unique loci in the genome. LncRNAs are responsible for transcriptional control of gene expression, both by site-specific recruitment of chromatin remodeling complexes (epigenetic changes) and by favoring or inhibiting the recruitment of transcription factors on defined promoters. These mechanisms may affect the expression timing of proteins, as well as of miRNAs, thus contributing to shape both the proteome and the miRNome of a developing cell. However, lncRNAs may exert their control also post-transcriptionally by driving molecular complexes responsible for editing, splicing, transport and degradation towards mRNA molecules. At the post-transcriptional level, lncRNAs will work alongside with miRNAs, thus contributing to establish a more flexible and fine-tuned system of regulation. This functional link, evident in adaptive immunity system, reveals a more general mechanism for the control of patterning and lineage commitment. Understanding how ncRNAs are regulated and exert their function in the context of the immune system will encourage further advancements on studies in this field and the exploitation of ncRNAs as possible candidates for future therapies.

## Figures and Tables

**Table 1:** miRNAs in adaptive immunity. HSC, hematopoietic stem cell; DN, double negative; DP, double positive; SP, single positive.

	MicroRNA	Expression	Targets	Function	Related alterations	References
**T-cell development**	miR-125b	↑ in HSC	Bak	Regulation of HSC compartment size	HSC exhaustion	[29–33]
	miR-181	↑ in DN and DP cells; ↓ in SP and mature T-cells	Dusp5, Dusp6, Shp2, Ptpn22	B-cell lineage differentiation	↑ in SLE	[34–36]
	miR-150	↑ DP CD8+ cells ↓ DN CD8+ cells	Notch3	T-cell development	↓in T-CLL	[20]
**B-cell development**	miR-17-92	↑ in progenitor B-cells; ↓ in mature B-cells	Pten, Bim	Defective central memory development	↑ B-cell lymphomas	[46,47]
	miR-150	↑ resting B-cells	cMyb	Impaired B1 cell maturation and Ab response	↓ B-cell lymphomas	[37,38]
	miR-34a	↓ pro-B lymphocytes	FoxP1	Required for pro-B to pre-B-cell transition	↑ B-cell lymphomas	[48]
	miR-155	↑ activated mature B-cells	Ship, C/EbpB, Hdac4	Impaired germinal center B-cell response	↑ diffuse large B-cell lymphoma	[13,49,50]
	miR15a-16	↑ CD5+ B-cells	Bcl2	Defect in apoptosis	↓ in B-CLL	[51]
**T-cell function**	miR-125b	↑ naïve CD4+ T-cells	Ifng, IL10Ra, IL2Rb, Prdm1	Differentiation of effector T-cells	↓ in SLE	[44]
	miR-182	↑ activated T-cells	Foxo1	Altered T-cell induced inflammation	Altered Treg mediated control of Th2 response	[43]
	miR155	↑ activated CD4+ T-cells	cMaf	Th lineage decisions: KO mice shows ↑ Th2 cells and ↓ Th1 and Th17 cells	↑ in SLE and RA	[13,52]
	miR-146a	↑ CD4+ and CD8+ memory cells	Irak1, Traf6, Fadd	Modulation of IL-2 production and AICD	↑ in RA and ↓ in SLE	[18,52–55]
	miR-17-92	↑ CD4+ and CD8+ memory cells	Pten, Bim	Defect in apoptosis	Lymphoproliferation	[46,56]
**Treg cells**	miR-10a	↑ Treg	Bcl6, Ncor2	Altered Th17 differentiation	Breakdown of peripheral tolerance	[57]
	miR-155	↑ Treg	Socs1	Altered Treg proliferation and homeostasis	Defect in Treg cell-mediated tolerance	[58–60]
	miR-146a	↑ Treg	Stat1	Altered Treg number, compromised suppressor activity	Autoimmunity due to altered Treg-mediated control of Th1 response	[61]

**Table 2:** lncRNAs in adaptive immunity.

	lncRNA	Genomic coordinates	Lenght	Expression	Characteristics	Proposed function	References
**has lncRNAs**	**Tmevpg1 (NeST)**	chr12: 68, 383, 225-68, 415, 107	32 kb	Activated CD8^+^ T cells CD4^+^ Th1 and Th17 cells	Its expression is regulated by Tbet and STAT4	Epigenetic control of Ifnγ locus	[103–105]
	**NRON**	chr9: 129, 170, 054-129, 172, 783	2.7 kb	CD4^+^ and CD8^+^ T cells	Sequence omology conserved back to chicken	Modulator of NFAT nuclear trafficking	[101]
**mmu lncRNAs**	**AK009498**	chr1: 58, 711, 508-58, 713, 886	710 bp	CD4^+^ and CD8^+^ effector and memory cells	Overlaps flip transcription start site	Potential negative regulator of Flip	[14]
	**Lef1as (AK029296)**	chr3: 131, 109, 026-131, 112, 090	3 kb	naive CD8^+^ T cells	Located antisense to Lef1 mRNA	Possible role in suppressionLef1	[14]
	**AK053349**	chr14: 115, 044, 497-115, 046, 726	2.2 kb	effector CD8^+^ T cells	Partially overlaps miR-17-92 cluster	unknown	[14]
	**AK020764**	chr11: 87, 755, 577-87, 757, 267	1.6 kb	effector CD8^+^ T cells	It hosts in its first intron miR-142	Possible functional link with miR-142	[14]
